# Heterogeneity of *Orientia tsutsugamushi* genotypes in field-collected trombiculid mites from wild-caught small mammals in Thailand

**DOI:** 10.1371/journal.pntd.0006632

**Published:** 2018-07-16

**Authors:** Ratree Takhampunya, Achareeya Korkusol, Sommai Promsathaporn, Bousaraporn Tippayachai, Surachai Leepitakrat, Allen L. Richards, Silas A. Davidson

**Affiliations:** 1 Department of Entomology, Armed Forces Research Institute of Medical Sciences-United States Army Medical Directorate, Bangkok, Thailand; 2 Viral and Rickettsial Diseases Department, Naval Medical Research Center, Silver Spring, Maryland, United States of America; University of Tennessee, UNITED STATES

## Abstract

Trombiculid mites are the vectors of scrub typhus, with infected larval mites (chiggers) transmitting the causative agent, *Orientia tsutsugamushi*, during feeding. Co-existence of multiple *O*. *tsutsugamushi* strains within infected mites has previously been reported in naturally infected, laboratory-reared mite lines using molecular methods to characterize the 56-kDa type-specific antigen (TSA) gene. In the current study, more advanced next-generation sequencing technology was used to reveal the heterogeneity of O. *tsutsugamushi* genotypes in field-collected trombiculid mites from rodents and small mammals in scrub typhus-endemic areas of Thailand. Twenty-eight trombiculid mites collected from 10 small mammals were positive for *O*. *tsutsugamushi*, corresponding to a prevalence rate of 0.7% within the mite population. Twenty-four of the infected mites were *Leptotrombidium* spp., indicating that this genus is the main vector for *O*. *tsutsugamushi* transmission in Thailand. In addition, *O*. *tsutsugamushi* was detected in the mite genera *Ascoschoengastia*, *Blankaartia*, *Gahrliepia*, and *Lorillatum*. Of the 10 infested small animal hosts, six had 2–10 infected mites feeding at the time of collection. Deep sequencing was used to characterize mixed infections (two to three *O*. *tsutsugamushi* genotypes within an individual mite), and 5 of the 28 infected mites (17.9%) contained mixed infections. Additionally, 56-kDa TSA gene sequence analysis revealed identical bacterial genotypes among co-feeding mites with single or mixed infections. These results suggest that co-feeding transmission may occur during the feeding process, and could explain the occurrence of mixed infections in individual mites, as well as the recovery of multiple infected mites from the same host. This study also revealed highly diverse within-host *O*. *tsutsugamushi* genotypes. The occurrence of multiple *O*. *tsutsugamushi* genotypes within individual mites has important implications, and could provide a mechanism for pathogen evolution/diversification in the mite vector.

## Author summary

Scrub typhus is a leading cause of undifferentiated febrile illness, putting 1 billion people at risk of infection. Trombiculid mites are the major vectors of the causative agent of scrub typhus, *Orientia tsutsugamushi* (OT), which is transferred through the bite of an infected mite. Previously, we reported the co-existence of multiple OT strains within laboratory-reared mites. Here, we extended our investigation into wild trombiculid mite populations collected from areas of Thailand where scrub typhus is endemic. More advanced next-generation sequencing (NGS) technology was used to examine the presence and abundance of mixed infection in individual mites based on sequence analysis of the 56-kDa type-specific antigen gene. NGS data revealed heterogeneity of OT genotypes in field-collected mites, and mixed infection with two to three OT genotypes was consistently observed (prevalence rate = 17.9%). Moreover, the identical OT genotype detected among co-feeding mites with single or mixed infections suggesting that co-feeding transmission may occur during the feeding process. This result could explain the occurrence of mixed infections in individual mites, as well as the recovery of multiple infected mites from the same host.

## Introduction

Scrub typhus is caused by intracellular Gram-negative bacteria belonging to the genus *Orientia*, although *O*. *tsutsugamushi* was thought to be the only causative agent until recently. *Orientia* species have been separated from the genus *Rickettsia* on the basis of differences in genome arrangement and cell wall structure, as well as the substantial genetic distance between the two genera [1, 2]. Recently, the incidence of scrub typhus has drastically increased in several tropical and sub-tropical countries, including Bhutan, Nepal, and Thailand [3, 4]. Environmental changes such as deforestation, urbanization, and even natural disasters have been suggested to play crucial roles in this reemergence [5–7]. In addition, new species of *Orientia* and the presence of the pathogen in locations outside of the previously described endemic region of the Tsutsugamushi triangle have been documented [8]. Recent evidence suggests the emergence of new *Orientia* species in both the Middle East and South America, with infected patients presenting with symptoms similar to those of scrub typhus [9, 10]. Serology testing of patient-paired serum (acute and convalescent) revealed four-fold increases in antibody titers against *O*. *tsutsugamushi* antigens in these patients. Comparison of the 16S rRNA, 47-kDa HtrA, and 56-kDa type-specific antigen (TSA) gene sequences against the GenBank database showed that the causative agents were most closely related to, but clearly separated from, *O*. *tsutsugamushi*. Based on these findings, one of the isolates was proposed as a new species of *Orientia*, and named *Candidatis* Orientia chuto [10].

Currently, there is no vaccine available for scrub typhus infection. The prophylactic treatment with antibiotic as a prevention method is recommended by World Health Organization (WHO) under special circumstances in endemic areas. Other general protective measures include avoiding exposure conditions, wearing appropriate clothing, and using insect and spatial repellents to prevent the chigger bites [11, 12]. Patients initially present with non-specific flu-like symptoms such as fever, rash, headache, myalgia, cough, generalized lymphadenopathy, nausea, vomiting, and abdominal pain approximately 5–14 days after being bitten by an infected mite [13, 14]. The fatality rate can be as high as 50% among these patients if left untreated. An eschar at the bite site is a feature of scrub typhus disease; however, the prevalence of eschar formation varies from 1–97% depending on the geographical area [15–17]. Trombiculid mites are the primarily vectors of *O*. *tsutsugamushi*, especially species belonging to the genus *Leptotrombidium*, including *L*. *arenicola*, *L*. *deliense*, *L*. *pallidum*, *L*. *fletcheri*, *L*. *scutellare*, *L*. *chiangraiensis*, *L*. *imphalum*, and *L*. *akamushi* [18–20]. In South Korea and Japan, *L*. *pallidum* and *L*. *scutellare* are the predominant vector species, and are distributed throughout the islands [21–23], while in Taiwan and Thailand, *L*. *deliense* is the main vector [24, 25]. Other mite genera are also recognized as potential vectors of scrub typhus, including *Blankaartia* and *Ascoschoengastia*; however, *O*. *tsutsugamushi* carriage has not been as deeply investigated in these genera [26, 27]. In general, mite species known to be important or potential vectors of *O*. *tsutsugamushi* are widely distributed throughout Asia, northern Australia, and the western Pacific Islands, placing an estimated one billion people at risk of scrub typhus [28].

To assess the diversity of *O*. *tsutsugamushi* isolates serotyping was developed utilizing serum raised in laboratory animals to individual orientia isolates. The produced antiserum or antisera reactive to new orientia isolates by IFA indicated how closely related the new isolate was to known isolates used to produce the antisera and consequently the lack of reactivity indicated the new isolate was antigenically distinct from the isolate(s) that produced the sera. Early on three prototypes (antigenically distinct isolates) strains were identified, Karp, Kato and Gilliam. Subsequently other antigenically distinct isolates were identified. Utilizing the serotyping technique it was found that Karp was the most prevalent *O*. *tsutsugamushi* serotype of isolates found in rodents and vectors in Thailand, with other serotypes appearing to be less common [29].

With advances in genetic characterization of bacteria the relationship between serotyping and genotyping of orientia isolates was assessed. Early studies showed that serotyped *O*. *tsutsugamushi* isolates using hyperimmune serum raised against prototype strains of the genotypes (genetically distinct) Karp, Gilliam, Kato, and TA716, resulted in cross-reactivity against genetically diverse isolates suggesting that many serotypes existed [30, 31]. Interestingly, the Gilliam and Japanese Gilliam serotypes suggested that these orientia isolates were closely related antigenically, however with genotyping based on the 56-kDa TSA gene they were shown to be genetically disparate (25). Thus, the genotyping method based on the 56-kDa TSA gene sequence and subsequent phylogenetic analysis, revealed substantial genotypic diversity among *O*. *tsutsugamushi* isolates from patients and rodent hosts in many parts of Thailand contrary to the serotyping study just mentioned above and that many of these new isolates were found to have much less similarity to the prototype strains than previously thought using serotyping techniques [25, 32, 33].

*O*. *tsutsugamushi* is maintained through both transovarial and transstadial transmission processes in the mite population, with these mechanisms thought to be the main ways in which the bacterium is maintained in the wild [20, 34–36]. Nevertheless, a small number of studies have reported the acquisition of *O*. *tsutsugamushi* from an infected host to naïve mites, although the bacteria failed to transmit transovarially [37–39]. Moreover, co-feeding transmission of *O*. *tsutsugamushi* from infected mite(s) to co-feeding naïve mites of the same or different species has been demonstrated in a laboratory setting; however, the study did not confirm whether this type of infection resulted in subsequent transstadial and transovarial transmission [40]. In ticks, co-feeding transmission of pathogens such as viruses occurs when infected individuals co-fed with uninfected individuals [41–44]. This direct tick-to-tick mode of transmission is thought to be an important process in maintaining viruses in nature. In addition, the bacterial pathogens responsible for Lyme disease, *Borrelia burgdorferi* and *Borrelia afzelii*, can be transmitted from infected nymphal *Ixodes ricinus* L. ticks to co-fed uninfected larval ticks [45, 46]. Although co-feeding transmission of these bacterial spirochetes is thought to occur in nature, albeit to a lesser extent than was observed in the laboratory, the perpetuation of the bacteria in nature largely depends on acquisition from systemically-infected hosts. Co-feeding transmission of *Rickettsia* has been observed among ticks, with uninfected *Rhipicephalus sanguineus* ticks being much more likely to acquire a *R*. *conorii* infection when feeding in close proximity to infected ticks, compared to feeding on an infected animal host [47]. A recent study has also demonstrated the co-feeding transmission of *Rickettsia felis* from infected cat fleas (*Ctenocephalides felis*) to naïve cat fleas or rat fleas (*Xenopsylla cheopis*) on a vertebrate host, and this mode of transmission could be crucial for the maintenance of *R*. *felis* within the vector population [48]. Although the occurrence of horizontal transmission and co-feeding transmission of *O*. *tsutsugamishi* has not been well documented, this mode of transmission might play an important role in maintaining *O*. *tsutsugamushi* in nature.

Our previous study showed the co-existence of two *O*. *tsutsugamushi* genotypes in single laboratory-reared mite lines [35]. Twelve colonies from three species of *Leptotrombidium* mites (*L*. *chiangraiensis* (Lc), *L*. *imphalum* (Li), and *L*. *deliense* (Ld)) were studied. An *O*. *tsutsugamushi* Karp-like genotype was found as a single genotype in the *L*. *chiangraiensis* line, but as part of a co-infection with an UT302-like genotype in *L*. *imphalum* lines or with a Gilliam-like genotype in the *L*. *deliense* line. Moreover, the co-existing genotypes were well maintained through transovarial and transstadial transmission in *L*. *imphalum* mites [36]. Co-infection with multiple *O*. *tsutsugamushi* sequence types was observed in 25% of patients in a study conducted by Sonthayanon et al. [49]. The study, along with several others, also identified a high degree of genetic diversity within the *O*. *tsutsugamushi* isolates recovered from patients [49–51]. Indeed, the genome of *O*. *tsutsugamushi* shows a high level of plasticity, with 50% of the genome containing repetitive sequences derived from integrative and conjugative elements. Amongst these, several hundred transposases, phage integrases, and transposable elements have been identified, as well as a massive duplication of components of the conjugative type IV secretion system (359 *tra* genes), which is distributed throughout the genome [52, 53]. These findings indicate that genetic recombination, duplication, and rearrangement are major mechanisms driving *O*. *tsutsugamushi* genomic diversity and complexity. As *O*. *tsutsugamushi* is an obligate intracellular parasite, recombination would occur during coinfection of different bacterial strains within the same mammalian host or vector, resulting in the exchange of genetic material between the bacterial strains. The co-existence of *O*. *tsutsugamushi* strains of different genotypes in a single trombiculid mite is confirmation that these mechanisms are likely to contribute to the genomic diversity/complexity of the bacterial pathogen.

In this study, we aimed to examine the prevalence of mixed infection of *O*. *tsutsugamushi* genotypes in individual field-collected mites in high and low scrub typhus-endemic areas of Thailand. Next generation sequencing (deep sequencing) of the 56-kDa TSA gene was used to accurately determine the presence and abundance of each *O*. *tsutsugamushi* genotype in individual infected mites. Additionally, the evidence for horizontal transmission of *O*. *tsutsugamushi* between co-feeding mites was discussed based on the study data. The results presented in this study represent an epidemiological assessment of *O*. *tsutsugamushi* carriage in a wild vector population in an area with active scrub typhus transmission.

## Methods

### Study sites and trombiculid mite collection

Rodents and mites were collected during the wet season (Jun–Aug) in the northeastern (Sisaket and Loei provinces), western (Tak Province), and southern (Pang Nga and Chumphon provinces) regions of Thailand in 2015 (Table 1). All study sites were on private land, and permission was obtained from each of the owners to conduct research on their land. None of the field studies involved endangered or protected species. Rodents were captured using live traps baited with bananas, palm fruit, or dried fish, and were collected from orchards, palm and rubber plantations, cultivated rice-fields, grassland areas, edges of dense forest, stream margins, and around dwellings. Traps were set for 3–5 nights and were checked early in the morning. Captured rodents were removed from the traps, euthanized using carbon dioxide, and processed immediately at the site of collection. Blood, serum, and tissue samples (liver, spleen, kidney, and lung) were collected and stored on dry ice. Ears were removed and stored in 70% ethanol for mite collection. All tissues were then transported to the AFRIMS laboratory for further processing. All rodents were later identified to the species level as described previously [54]. A recent genetic analysis of rodents in Southeast Asia showed that the black rat, *Rattus rattus* sensu stricto, is not found in Thailand. However, three morphologically similar species are present; *Rattus tanezumi*, *Rattus sakeratensis*, and an additional mitochondrial lineage of unclear taxonomic status referred to as ‘Rattus R3’ [55]. These three species were not separated in this study using molecular methods and are referred to collectively as *Rattus rattus* complex. Meanwhile, all other members of the genus *Rattus* are identified to species.

**Table 1:** Prevalence of *Orientia tsutsugamushi* in rodents, small mammals, and associated trombiculid mites captured from June–August 2015 from five provinces in three regions of Thailand.

Location	GPS coordinates(latitude/longitude)	No. of *O*. *tsutsugamushi-*infected animals/total no. animals captured (% infection)	No. of *O*. *tsutsugamushi*-infected mites/total no. mites collected (% infection)	No. of *O*. *tsutsugamushi*-infected mites/total no. mites collected (% infection)						
				*Ascoschoengastia*	*Blankaartia*	*Eutrombicula*	*Gahrliepia*	*Leptotrombidium*	*Lorillatum*	*Schoengastia*
**Tak**	**16.401034/98.749251**	**0/91 (0)**	**0/654 (0)**	**0/127 (0)**	**0**	**0**	**0/522 (0)**	**0/5 (0)**	**0**	**0**
**Loei**	**17.351113/101.15821 **	**0/14 (0)**	**0/150 (0)**	**0/68 (0)**	**0**	**0**	**0/81 (0)**	**0/1 (0)**	**0**	**0**
**Sisaket**	** 14.477182/104.489909**	**0/29 (0)**	**2/613 (0.3)**	**1/344 (0.3)**	**0**	**0**	**0/156 (0)**	**0**	**1/113 (0.9)**	**0**
**Chumphon**	**10.578439/99.212795 **	**0/75 (0)**	**0/1605 (0)**	**0/325 (0)**	**0/32 (0)**	**0**	**0/114 (0)**	**0/1124 (0)**	**0**	**0/10 (0)**
**Phang Nga**	**8.664258/98.452195 **	**3/66 (4.6)**	**26/1259 (2.1)**	**0/8 (0)**	**1/29 (3.5)**	**0/9 (0)**	**1/170 (0.6)**	**24/972 (2.5)**	**0/46 (0)**	**0/25 (0)**
**Total **		**3/275 (1.1)**	**28/4281 (0.7)**	**1/872 (0.1)**	**1/61 (1.6)**	**0/9 (0)**	**1/1043 (0.1)**	**24/2102 (1.1)**	**1/159 (0.6)**	**0/35 (0)**

The animals were collected from Tak Province (western region), Loei and Sisaket provinces (northeastern region), and Chumphon and Pang Nga provinces (southern region). The prevalence (%) of *O*. *tsutsugamushi* in both animals and chiggers recovered from the animal hosts was determined.

### Genomic DNA extraction from rodent tissue

Genomic DNA was extracted from rodent spleen samples using a Wizard Genomic DNA Purification Kit (Promega, Madison, WI) according to the manufacturer’s instructions, with some modifications as per a previously published protocol [56]. Briefly, spleen tissue was cut into pieces (~3 mm in diameter) and added to 600 μl of Nuclei Lysis Solution (Promega, Madison, WI). The mixture was homogenized with beads using a TissueLyser II apparatus (Qiagen, Hilden, Germany) at 25 Hz for two rounds of 5 min each. The homogenized solution was then incubated with 20 μl of Proteinase K solution (20 mg/ml) at 55°C for 1 h, and then with 3 μl of RNase A (10 mg/ml) at 37°C for 15 min. A 200-μl volume of protein precipitation solution was then added, and the sample was mixed vigorously by vortex then incubated on ice for 5 min. Insoluble materials were removed by centrifugation at 20,000 x g for 4 min, and the supernatant was transferred to a new tube. DNA was precipitated by adding 600 μl of isopropanol, followed by centrifugation at 20,000 x g for 1 min. The resulting DNA pellet was washed with 70% ethanol and then air dried. Dried DNA was resuspended in 200 μl of EB buffer (10 mM Tris Cl, pH 8.5) and stored at −20°C until further analysis.

### Mite morphological identification and DNA extraction

Mites in their larval stage (chigger) were collected from rodent ears by paintbrush under the stereomicroscope and stored separately. Each mite was individually identified to the genus level under a high resolution microscope (400×, Nikon ECLIPSE Ni-U microscope, Tokyo, Japan) using a taxonomic key [57]. Mites were not identified to the species level because this would have required the mites to be cleared and slide-mounted, which would prevent molecular testing. The genus of each mite was recorded, along with its host species and other field site information. Mites collected from wild-caught rodents were individually subjected to genomic DNA extraction using a modified tissue protocol from the QIAamp DNA Mini Kit (Qiagen) with a previously published protocol [35]. Mites were punctured with a fine needle under stereomicroscope to release the tissue from the hard chitin exoskeleton prior to DNA extraction. Eluted DNA solution was stored at −20°C until further use in *O*. *tsutsugamushi* quantitative polymerase chain reaction (qPCR) screening assays. Chigger cytochrome oxidase subunit 1 (host COI gene) was examined by conventional PCR on all mite samples before being subjected to Otsu47 qPCR assay as describe previously [58].

### Screening of *O*. *tsutsugamushi* from field-collected trombiculid mites and animal tissue

Field-collected mites and animal tissue (spleen) samples were screened for the presence of *O*. *tsutsugamushi* by qPCR analysis. The primers and probe were designed to detect a portion of the 47-kDa HtrA gene [59]. The reaction mixtures (25 μl) contained 12.5 μl of 2× Platinum Quantitative PCR SuperMix-UDG (Invitrogen, Foster City, CA), 0.2 mM probe, and 0.2 mM each primer. The qPCR assay, referred to as Otsu47, was performed by incubating samples at 95°C for 2 min, followed by 45 cycles of 95°C for 15 s and 60°C for 1 min. Reactions were carried out using the ABI 7500 Fast Real-time PCR System (Life Technologies, Carlsbad, CA).

### Amplification of the 56-kDa TSA gene for next-generation sequencing

Trombiculid mites or animal tissue found to be positive for the presence of *O*. *tsutsugamushi* was then used as the basis for amplification and next-generation sequencing (NGS) of the 56-kDa TSA gene from *O*. *tsutsugamushi*. A fragment of the 56-kDa TSA gene (variable domains I–III, 600–700 bp) was amplified in a first-round PCR using primers RTS-8 and RTS-9 [60]. The reaction was performed in a 20-μl volume containing 5 μl of DNA template, 300 nM each primer, 200 μM dNTPs, 1.5 mM MgCl_2_, 1× PCR buffer, and 0.4 U of iProof High-Fidelity DNA Polymerase (Bio-Rad, Hercules, CA). Amplification was performed using a DNA thermal cycler under the following conditions: initial denaturation at 98°C for 2 min; 30 cycles of 98°C for 10 s, 45°C for 20 s, and 72°C for 45s; and a final extension at 72°C for 10 min. The second amplification was performed using fusion primers for NGS. The fusion primers comprised three parts: (i) 21-bp A and B sequencing adapters plus 4 bp of key sequence (bold letters) (5′-CGTATCGCCTCCCTCGCGCCA**TCAG**-3′ and 5′-CTATGCGCCTTGCCAGCCCGC**TCAG**-3′, respectively), (ii) 10-bp multiplex identifier (MID) sequences (with a unique MID for each sample), and (iii) 56-kDa TSA gene-specific sequences RTS-6 and RTS-7 [60]. PCR assays were carried out in 50-μl reaction volumes containing 1.5 mM MgCl_2_, 200 μM dNTPs, 0.3 μM each primer, and 0.5 U of iProof High-Fidelity DNA Polymerase. Five microliter of diluted product from the first amplification (1:50) was used as template. The thermal cycler parameters consisted of 98°C for 2 min, followed by 30 cycles of 98°C for 10 s, 55°C for 30 s, and 72°C for 30 s. The resulting amplicons were purified using AMPure beads (Agencourt Bioscience Corporation, Beverly, MA), and amplicon concentrations were measured using the Quant-iT PicoGreen dsDNA Assay (Invitrogen) according to the manufacturer’s protocol. Each PCR amplicon was then diluted to 10^9^ copies/μl and pooled (10–15 amplicons per pool). Sample pools were amplified by emulsion PCR using a GS Junior+ emPCR Kit (Lib-A) with a ratio of 0.5 copies per bead. Amplicons were then sequenced in both forward and reverse directions at 5000× coverage per sample on a 454 GS Junior Genome Sequencer Instrument using the GS Junior+ Sequencing Kit XL+ chemistry (Roche 454 Life Sciences, Branford, CT). The average read length obtained from the NGS runs was 633 nucleotides, with a median number of reads per sample of 10,198, (range 3,749–28,943).

### Data analysis

Raw reads were quality filtered and de-multiplexed with standard quality filtering parameters using CLC Genomics Workbench v 9.0 software (Qiagen). After quality trimming, reads < 500 bp and > 800 bp in length were discarded. All trimmed reads were then de-multiplexed using the MID barcodes. All reads from each sample were mapped to a full reference set (S1 Table) using the Map Reads to Reference function in the CLC Genomics Workbench software. Each read was mapped to the reference sequences for which it showed the best match using the parameters length fraction = 0.5 and similarity fraction = 0.8 (at least 50% of the total alignment and 80% identity), then the reads were counted and extracted from each reference. In order to be assigned to a *O*. *tsutsugamushi* genotype, the number of reads aligned to each reference had to be at least 5% of the total reads. The extracted reads per reference were assembled using de novo assembly, and the consensus sequence was extracted. The GenBank accession numbers for consensus sequences reported in this study were provided at the end of this article. The consensus sequences were aligned with reference sequences retrieved from the GenBank database using the MUSCLE codon alignment algorithm [61]. A maximum likelihood phylogenetic tree was then constructed based on the 56-kDa TSA gene variable domain I–III sequences using the GTR+G model of nucleotide substitution with bootstrapping (1000 replicates) in MEGA 6 [62] (S1 Fig).

### Characterization of *O*. *tsutsugamushi* genotypes by cloning

The 56-kDa TSA gene was amplified by nested polymerase chain reaction using our previously designed primer [35] for the first-round PCR, and then nested PCR was carried out as previously described [63]. The resulting 615-678-bp amplicon was purified using a Qiagen DNA Purification Kit to remove salts and primer dimers. The purified fragment was then cloned into pCR2.1-TOPO and transformed into *Escherichia coli* DH5a-T1^R^ as per the manufacturer’s instructions (Invitrogen). Transformants were randomly selected and screened by nested PCR. Between 6 and 28 clones from individual chiggers appeared to contain the correct 56-kDa TSA gene insert fragment. Plasmids containing the correct 56-kDa TSA gene insert were then purified from the *E*. *coli* host using a QIAprep Spin Miniprep Kit (Qiagen), and sent for DNA sequencing (Sanger method) by AITBiotech (Singapore).

### Statistical analysis and data visualization

All statistical analyses (Chi-square tests) and graphical illustrations presented in this study were performed in the R environment for statistical computing [64–66]. A nucleotide distance matrix was generated using “DNADist DNA Distance Matrix” in BioEdit [67]. A heatmap dendrogram was generated in R using the heatmap.2 function from the package *Gplots* and applying complete linkage clustering and Euclidean distances computed among 36 genotypes from 28 infected mites [68].

### Ethics statement

Rodents were trapped according to the institutional animal collection protocol titled “Field Sampling of Small Mammal (Orders: *Erinaceomorpha*, *Soricomorpha*, *Scandentia*, *Macroscelidea*, and *Rodentia*) Populations to Support Zoonotic Diseases Surveillance and Ectoparasite Collection” (PN# 12–06), reviewed and approved by the USAMC-AFRIMS Institutional Animal Care and Use Committee (IACUC). All sampling procedures and experimental manipulations were reviewed and approved as part of obtaining the animal collection protocol (PN# 12–06). Research was conducted in compliance with the Animal Welfare Act and other federal statutes and regulations relating to animals and experiments involving animals, and adhered to principles outlined in the Guide for the Care and Use of Laboratory Animals, NRC Publication, 2011 edition.

### Accession numbers

The GenBank (http://www.ncbi.nlm.nih.gov/Genbank/) accession numbers for the 56-kDa TSA gene described in this paper are: MH290189 (Lep.DS092.9c), MH290190 (Lep.DS092.8c), MH290191 (Lep.DS021.b), MH290192 (Lep.DS016.b), MH290193 (Lep.DS092.6b), MH290194 (Lep.DS020.2a), MH290195 (Lep.DS092.8a), MH290196 (Lep.DS021.1h), MH290197 (Lep.DS016.e), MH290198 (Lep.DS092.8e), MH290199 (Lep.DS092.9e), MH290200 (Lep.DS092.10e), MH290201 (Lep.DS123.1e), MH290202 (Lep.DS078.d), MH290203 (Lep.DS021.3f), MH290204 (Lep.DS092.7g), MH290205 (Lep.DS092.5g), MH290206 (Lep.DS092.4g), MH290207 (Lep.DS092.2g), MH290208 (Lep.DS092.1g), MH290209 (Lep.DS020.1g), MH290210 (Lep.DS021.4g), MH290211 (Lep.DS123.2g), MH290212 (Lep.DS027.g), MH290213 (Lep.DS030.g), MH290214 (Asc.MS651.g), MH290215 (Lep.DS024.b), MH290216 (Bla.DS123.a), MH290217 (Lep.DS092.3e), MH290218 (Bla.DS123.e), MH290219 (Lep.DS024.d), MH290220 (Lep.DS024.g), MH290221 (Bla.DS123.g), MH290222 (Gah.DS024.g), MH290223 (Lep.DS021.2g), and MH290224 (Lor.MS651.g).

## Results

### Abundance of small mammals and rodents in the study areas

Collection sites were selected based on our annual surveillance data (2012–2016) on scrub typhus prevalence in small mammals and trombiculid mites in Thailand (S2 Fig). The data revealed a high prevalence of *O*. *tsutsugamushi* in Chumphon and Pang Nga provinces (southern Thailand), while low prevalence rates were observed in Sisaket and Loei (northeastern Thailand) and Tak (western Thailand) provinces. Therefore, we decided to survey both high and low prevalence areas for comparison. Small mammals and rodents were collected during the rainy season (Jun–Aug) of 2015. In total, 275 small mammals and rodents were collected from five provinces in three regions of Thailand (Table 1). The collected animals belonged to eight genera and 15 species (Table 2). The greatest rodent species diversity was observed in Pang Nga Province, followed by Tak Province. *R*. *rattus* complex (n = 113), *Bandicota indica* (greater bandicoot rat, n = 54), and *Mus caroli* (ryukyu mouse, n = 42) were the most abundant species sampled in this study, accounting for 41.1%, 19.6%, and 15.3% of total rodents collected, respectively. *R*. *rattus* complex were the most abundant species in all provinces (34.5–65.3%) except Tak Province, in which *M*. *caroli* (46.2%) was the most abundant species collected.

**Table 2:** Chigger infestation rates and population diversity of mites recovered from small mammals, Thailand.

Host Species	No. of small mammals collected (% of total)	Small mammals infested with mite	No. of mites collected (% of total)	Chigger index (average no. mites/animal)	Mite genera (% of total)						
		(% infestation)			*Ascoschoengastia*	*Blankaartia*	*Eutrombicula*	*Gahrliepia*	*Leptotrombidium*	*Lorillatum*	*Schoengastia*
**Tak**	**91 (33.1)**	**30 (33.0)**	**654 (15.3)**	**7.2**	**127 (3.0)**	**0**	**0**	**522 (12.2)**	**5 (0.1)**	**0**	**0**
*Bandicota indica*	26 (28.6)	22 (84.6)	386 (9.0)	14.8	32 (0.8)	0	0	353 (8.3)	1 (0)	0	0
*Mus caroli*	42 (46.2)	0	0	0.0	0	0	0	0	0	0	0
*Mus cervicolor*	14 (15.4)	0	0	0.0	0	0	0	0	0	0	0
*Berylmys berdmorei*	1 (1.1)	1 (100)	28 (0.7)	28.0	28 (0.7)	0	0	0	0	0	0
*Rattus exulans*	1 (1.1)	0	0	0.0	0	0	0	0	0	0	0
*Rattus mackenziei*	1 (1.1)	1 (100)	2 (0.1)	2.0	0	0	0	2 (0.1)	0	0	0
*R*. *rattus* complex	6 (6.6)	6 (100)	238 (5.6)	39.7	67 (1.6)	0	0	167 (3.9)	4 (0.1)	0	0
**Loei**	**14 (5.1)**	**12 (85.7)**	**150 (3.5)**	**10.7**	**68 (1.6)**	**0**	**0**	**81 (1.9)**	**1 (0)**	**0**	**0**
*Rattus bukit*	4 (28.6)	4 (100)	42 (1.0)	10.5	2 (0.1)	0	0	39 (0.9)	1 (0)	0	0
*R*. *rattus* complex	9 (64.3)	8 (88.9)	108 (2.5)	12.0	66 (1.5)	0	0	42 (1.0)	0	0	0
*Tupaia glis*	1 (7.1)	0	0	0.0	0	0	0	0	0	0	0
**Sisaket**	**29 (10.5)**	**19 (65.5)**	**613 (14.3)**	**21.1**	**344 (8.0)**	**0**	**0**	**156 (3.6)**	**0**	**113 (2.6)**	**0**
*Crocidura horsfieldii*	1 (3.5)	0	0	0	0	0	0	0	0	0	0
*Berylmys berdmorei*	1 (3.5)	1 (100)	18 (0.4)	18	1 (0)	0	0	7 (0.2)	0	10 (0.2)	0
*Rattus bukit*	4 (13.8)	4 (100)	41 (1.0)	10.3	0	0	0	38 (0.9)	0	3 (0.1)	0
*Rattus exulans*	5 (17.2)	0	0	0	0	0	0	0	0	0	0
*R*. *rattus* complex	10 (34.5)	10 (100)	531 (12.4)	53.1	334 (7.8)	0	0	99 (2.3)	0	98 (2.3)	0
*Rattus surifer*	8 (27.6)	4 (50.0)	23 (0.5)	2.9	9 (0.2)	0	0	12 (0.3)	0	2 (0.1)	0
**Chumphon**	**75 (27.3)**	**65 (86.7)**	**1,605 (37.5)**	**21.4**	**325 (7.6)**	**32 (0.8)**	**0**	**114 (2.7)**	**1,124 (26.3)**	**0**	**10 (0.2)**
*Bandicota indica*	18 (24.0)	15 (83.3)	299 (7.0)	16.6	2 (0.1)	32 (0.8)	0	55 (1.3)	200 (4.7)	0	10 (0.2)
*R*. *rattus* complex	49 (65.3)	44 (89.8)	1135 (26.5)	23.2	319 (7.5)	0	0	58 (1.4)	758 (17.7)	0	0
*Rattus sabanus*	1 (1.3)	1 (100)	9 (0.2)	9.0	4 (0.1)	0	0	1 (0)	4 (0.1)	0	0
*Tupaia glis*	7 (9.3)	5 (71.4)	162 (3.8)	23.1	0	0	0	0	162 (3.8)	0	0
**Phang Nga**	**66 (24.0)**	**50 (75.8)**	**1,259 (29.4)**	**19.1**	**8 (0.2)**	**29 (0.7)**	**9 (0.2)**	**170 (4.0)**	**972 (22.7)**	**46 (1.1)**	**25 (0.6)**
*Bandicota indica*	10 (15.2)	8 (80.0)	302 (7.1)	30.2	0	24 (0.6)	0	5 (0.1)	268 (6.3)	4 (0.1)	1 (0)
*Chiropodomys gliroides*	1 (1.5)	0	0	0	0	0	0	0	0	0	0
*Menetes berdmorei*	3 (4.6)	2 (66.7)	20 (0.5)	6.7	0	4 (0.1)	4 (0.1)	0	12 (0.3)	0	0
*Rattus bukit*	4 (6.1)	0	0	0	0	0	0	0	0	0	0
*Rattus muelleri*	2 (3.0)	1 (50.0)	13 (0.3)	6.5	0	0	1 (0)	0	12 (0.3)	0	0
*R*. *rattus* complex	39 (59.1)	33 (84.6)	819 (19.1)	21	8 (0.2)	1 (0)	0	163 (3.8)	581 (13.6)	42 (1.0)	24 (0.6)
*Rattus sabanus*	2 (3.0)	2 (100)	44 (1.0)	22.0	0	0	0	0	44 (1.0)	0	0
*Tupaia glis*	5 (7.6)	4 (80.0)	61 (1.4)	12.2	0	0	4 (0.1)	2 (0.1)	55 (1.3)	0	0
**Total**	**275**	**176 (64.0)**	**4,281**	**15.6**	**872 (20.4)**	**61 (1.4)**	**9 (0.2)**	**1,043 (24.4)**	**2,102 (49.1)**	**159 (3.7)**	**35 (0.8)**

### Infestation rates and abundance and diversity of mites among small mammal and rodent populations, and geographical variation of mite genera among the study areas

**Fig 1:**
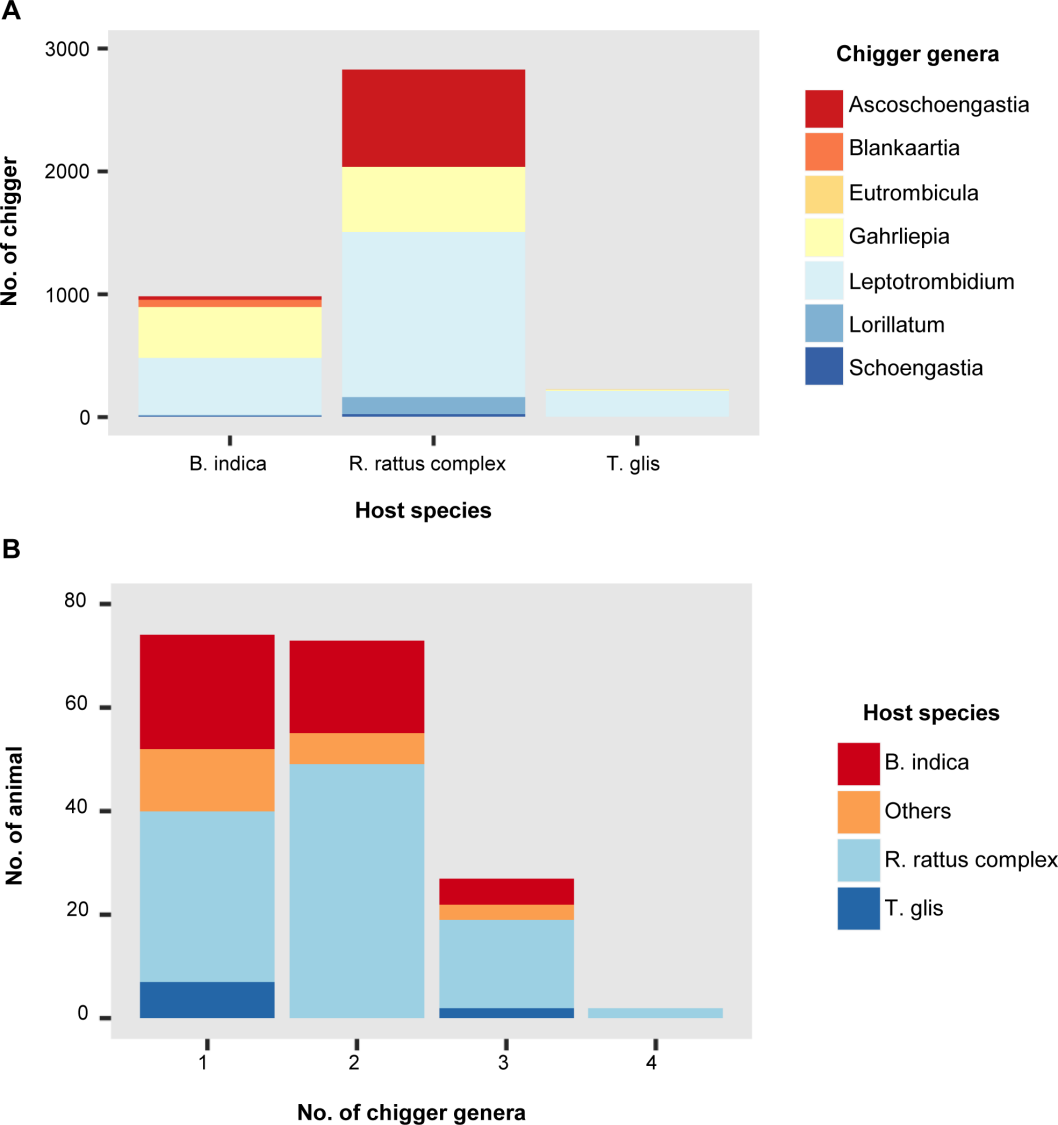
Abundance and diversity of field-collected trombiculid mites on wild-caught rodents and small mammals. (A) Abundance of chigger(s) from three dominant small mammal species. (B) Diversity of chigger(s) collected from animal(s).

A total of 4,281 trombiculid mites were collected from 176 of the 275 (64.0%) small mammals and rodents examined in this study (Table 2). The infestation rate by host species was determined for the most abundant species, with the highest infestation rates observed in *R*. *rattus* complex (101/113, 89.4%), *B*. *indica* (45/54, 83.3%), and *Tupaia glis* (common tree shrew; 9/13, 69.2%). Overall, mite infestation rates among animals collected from each of the provinces ranged from 33–86.7%. Of the infested animals, those from Sisaket, Chumphon, and Pang Nga provinces were heavily infested, with a chigger index (average number of chiggers per animal) ranging from 19.1–21.4 (Table 2). However, when present, the number of mites on individual hosts was highly variable (1–181). The most abundant mite genus collected from animals in this study was *Leptotrombidium* (49.1%), followed by *Gahrliepia* (24.4%) and *Ascoschoengastia* (20.4%). Overall, a significantly greater proportion of *Leptotrombidium* mites were collected relative to other mite genera (P < 0.001, Chi-square test), with the abundance of *Leptotrombidium* spp. clearly observed in Pang Nga and Chumphon provinces (southern Thailand) (Table 2). The greatest numbers of mites were collected from *R*. *rattus* complex (2,831, 66.1%), *B*. *indica* (987, 23.1%), and *T*. *glis* (223, 5.2%), with mean chigger index scores of 25.1 (2,831/113), 18.3 (987/54), and 12.2 (223/13), respectively (Fig 1A). However, when considering the proportion of *Leptotrombidium* spp. amongst all mite genera collected from these hosts, *T*. *glis* individuals showed the highest proportion of *Leptotrombidium* spp. mites (Chi-square test, P < 0.001). The majority of animals were infested with mites belonging to one (n = 74, 42%) or two (n = 73, 41.5%) different genera, while only 27 (15.3%) and two (1.1%) animals were infested with three or four different mite genera, respectively, with *R*. *rattus* complex accounting for the majority of these animals (19/29, 65.5%) (Fig 1B).

### Prevalence of *O*. *tsutsugamushi* in rodent and small mammal populations

The overall prevalence of *O*. *tsutsugamushi* in the animal populations was very low (1.1%, 3/275). The three infected rodents were identified as *R*. *rattus* complex (n = 2) and *B*. *indica* (n = 1), and were collected from Pang Nga Province (southern Thailand) (Table 1). However, trombiculid mites collected from the infected animals were all negative for *O*. *tsutsugamushi*. Genotyping of *O*. *tsutsugamushi* from these three rodents revealed single infections with Karp A-genotype strains (26–30 clones, S2 Table).

### Prevalence of *O*. *tsutsugamushi* in mite populations and genotyping by deep sequencing of the 56-kDa TSA gene (variable domains I–III)

Screening of the 4,281 individual mites for the presence of *O*. *tsutsugamushi* using the Otsu47 qPCR assay revealed positive results for 28 mites (prevalence = 0.7%). Twenty-four of the 28 infected mites were *Leptotrombidium* spp. (85.7%), while the remaining four mites were *Ascoschoengastia* spp., *Lorillatum* spp., *Gahrliepia* spp., and *Blankaartia* spp. (Table 3). The 28 trombiculid mites were collected from 10 animals: two mites were from one *R*. *rattus* complex individual collected in Srisaket Province, while the remaining 26 infected mites were from nine animals collected in Pang Nga Province (four *R*. *rattus* complex, three *B*. *indica*, and two *T*. *glis*). However, tissue samples from all 10 animals were negative for *O*. *tsutsugamushi* infection using the same assay. The overall infection rate among mites collected from these 10 animals was 8.4% (28/333), whereas the *O*. *tsutsugamushi* prevalence among mites per host varied from 2.2–21.3%. Prevalence rates for infection among the same mite species per host ranged from 0–21.3% (Table 3). The number of infected mites per host varied from 1–10, with four hosts having one infected mite and six hosts with more than two infected mites (2–10 infected mites).

**Table 3:** *Orientia tsutsugamushi*-positive rate in mite populations collected from 10 animals, Thailand.

No.	Host ID	Host species	Location	No. of infected mites/total collected by genera (% infection)							No. of infected mites/total collected (% infection)
				*Ascoschoengastia*	*Blankaartia*	*Eutrombicula*	*Gahrliepia*	*Leptotrombidium*	*Lorillatum*	*Schoengastia*	
1	MS0651	*R*. *rattus* complex	Sisaket	1/71 (1.4)	-	-	0/3 (0)	-	1/16 (6.3)	-	2/90 (2.2)
2	DS016	*R*. *rattus* complex	Pang Nga	-	-	-	-	1/20 (5.0)	-	0/1 (0)	1/21 (4.8)
3	DS020	*T*. *glis*	Pang Nga	-	-	0/1 (0)	0/1 (0)	2/14 (14.3)	-	-	2/16 (12.5)
4	DS021	*T*. *glis*	Pang Nga	-	-	0/3 (0)	0/1 (0)	5/25 (20.0)	-	-	5/29 (17.2)
5	DS024	*R*. *rattus* complex	Pang Nga	-	-	-	1/9 (11.1)	1/10 (10.0)	-	-	2/19 (10.5)
6	DS027	*R*. *rattus* complex	Pang Nga	-	0/1 (0)	-	-	1/20 (5.0)	-	-	1/21 (4.8)
7	DS030	*R*. *rattus* complex	Pang Nga	-	-	-	-	1/9 (11.1)	-	-	1/9 (11.1)
8	DS078	*B*. *indica*	Pang Nga	-	-	-	-	1/45 (2.2)	-	-	1/45 (2.2)
9	DS092	*B*. *indica*	Pang Nga	-	-	-	-	10/47 (21.3)	-	-	10/47 (21.3)
10	DS123	*B*. *indica*	Pang Nga	-	1/10 (10.0)	-	-	2/26 (7.7)	-	-	3/36 (8.3)
**No. of infected mites/total collected (% infection)**				1/71 (1.4)	1/11 (9.1)	0/4 (0)	1/14 (7.1)	24/216 (11.1)	1/16 (6.3)	0/1 (0)	28/333 (8.4)

Genotyping of *O*. *tsutsugamushi* was conducted for the 28 positive mites by NGS. Of these, the 56-kDa TSA gene was successfully amplified and sequenced from 21 mites using the NGS method. The target gene from the remaining seven mites was genotyped by cloning, as shown in Table 4. The genotypic characterization was based on 56-kDa TSA gene sequence identity to reference sequences in the database, as well as phylogenetic analysis (S1 Table and S1 Fig). Overall, eight *O*. *tsutsugamushi* genotypes were detected in the mite population. The majority of infected mites showed single infection (23/28, 82.1%), with only five mites (5/28, 17.9%) showing mixed infection with 2–3 different *O*. *tsutsugamushi* genotypes. The most prevalent *O*. *tsutsugamushi* genotype found in the mites was TA763 B (44.4%), followed by Kato B (19.4%) (Table 4). The majority of single infections (n = 23) were identified as genotype TA763 B strains (14/23, 60.9%), followed by Kato B (3/23, 13.0%) and Karp A (2/23, 8.7%). The remaining four single infections were identified as genotypes TA763 A, Kato A, and Gilliam JG-C, as well as an unknown genotype. Twenty of the 23 infected mites (86.9%) with single infection were *Leptotrombidium* spp., while the remaining mites were *Gahrliepia* spp., *Ascoschoengastia* spp., and *Lorillatum* spp.

**Table 4:** Genotyping of *Orientia tsutsugamushi* from individual mites by next-generation sequencing.

No.	Host ID	Host species	Mite genus	Location	Chigger ID	*O*. *tsutsugamushi* genotype No. of reads or clones* (% abundance)								Infection type
						Gilliam (a)	Karp A (b)	Karp C (c)	Kato A (d)	Kato B (e)	TA763 A (f)	TA763 B (g)	Unknown (h)	
1	MS0651	*R*. *rattus* complex	*Ascoschoengastia*	Sisaket	Asc.MS0651	-	-	-	-	-	-	5461 (100)	-	Single
2	MS0651	*R*. *rattus* complex	*Lorillatum*	Sisaket	Lor.MS0651	-	-	-	-	-	-	21* (100)	-	Single
3	DS016	*R*. *rattus* complex	*Leptotrombidium*	Pang Nga	Lep.DS016	-	3810 (52)	-	-	9083 (48)	-	-	-	**Mixed**
4	DS020	*T*. *glis*	*Leptotrombidium*	Pang Nga	Lep.DS020.1	-	-	-	-	-	-	4,092 (100)	-	Single
5	DS020	*T*. *glis*	*Leptotrombidium*	Pang Nga	Lep.DS020.2	21,935 (100)	-	-	-	-	-	-	-	Single
6	DS021	*T*. *glis*	*Leptotrombidium*	Pang Nga	Lep.DS021.1	-	-	-	-	-	-	-	10,466 (100)	Single
7	DS021	*T*. *glis*	*Leptotrombidium*	Pang Nga	Lep.DS021.2	-	-	-	-	-	-	14* (100)	-	Single
8	DS021	*T*. *glis*	*Leptotrombidium*	Pang Nga	Lep.DS021.3	-	-	-	-	-	6,474 (100)	-	-	Single
9	DS021	*T*. *glis*	*Leptotrombidium*	Pang Nga	Lep.DS021.4	-	-	-	-	-	-	17,554 (100)	-	Single
10	DS021	*T*. *glis*	*Leptotrombidium*	Pang Nga	Lep.DS021.5	-	6,270 (100)	-	-	-	-	-	-	Single
11	DS024	*R*. *rattus* complex	*Gahrliepia*	Pang Nga	Gah.DS024	-	-	-	-	-	-	28* (100)	-	Single
12	DS024	*R*. *rattus* complex	*Leptotrombidium*	Pang Nga	Lep.DS024	-	1* (9)	-	2* (18)	-	-	8* (73)	-	**Mixed**
13	DS027	*R*. *rattus* complex	*Leptotrombidium*	Pang Nga	Lep.DS027	-	-	-	-	-	-	14* (100)	-	Single
14	DS030	*R*. *rattus* complex	*Leptotrombidium*	Pang Nga	Lep.DS030	-	-	-	-	-	-	12,249 (100)	-	Single
15	DS078	*B*. *indica*	*Leptotrombidium*	Pang Nga	Lep.DS078	-	-	-	10,198(100)	-	-	-	-	Single
16	DS092	*B*. *indica*	*Leptotrombidium*	Pang Nga	Lep.DS092.1	-	-	-	-	-	-	3,749 (100)	-	Single
17	DS092	*B*. *indica*	*Leptotrombidium*	Pang Nga	Lep.DS092.2	-	-	-	-	-	-	9,900 (100)	-	Single
18	DS092	*B*. *indica*	*Leptotrombidium*	Pang Nga	Lep.DS092.3	-	-	-	-	6* (100)	-	-	-	Single
19	DS092	*B*. *indica*	*Leptotrombidium*	Pang Nga	Lep.DS092.4	-	-	-	-	-	-	5,214 (100)	-	Single
20	DS092	*B*. *indica*	*Leptotrombidium*	Pang Nga	Lep.DS092.5	-	-	-	-	-	-	14,598 (100)	-	Single
21	DS092	*B*. *indica*	*Leptotrombidium*	Pang Nga	Lep.DS092.6	-	9,933 (100)	-	-	-	-		-	Single
22	DS092	*B*. *indica*	*Leptotrombidium*	Pang Nga	Lep.DS092.7	-	-	-	-	-	-	28,943 (100)	-	Single
23	DS092	*B*. *indica*	*Leptotrombidium*	Pang Nga	Lep.DS092.8	3,143 (74)	-	814 (19)	-	307 (7)	-	-	-	**Mixed**
24	DS092	*B*. *indica*	*Leptotrombidium*	Pang Nga	Lep.DS092.9	-	-	374 (7)	-	4668 (93)	-	-	-	**Mixed**
25	DS092	*B*. *indica*	*Leptotrombidium*	Pang Nga	Lep.DS092.10	-	-	-	-	19,187 (100)	-	-	-	Single
26	DS123	*B*. *indica*	*Blankaartia*	Pang Nga	Bla.DS123	9* (38)	-	-	-	2* (8)	-	13* (54)	-	**Mixed**
27	DS123	*B*. *indica*	*Leptotrombidium*	Pang Nga	Lep.DS123.1	-	-	-	-	15,483(100)	-	-	-	Single
28	DS123	*B*. *indica*	*Leptotrombidium*	Pang Nga	Lep.DS123.2	-	-	-	-	-	-	10,258 (100)	-	Single

All mixed *O*. *tsutsugamushi* genotype infections (n = 5) were identified in animals from Pang Nga Province. Four infected mites were *Leptotrombidium* spp., and one was *Blankaartia* spp. Two *Leptotrombidium* mites had a mixed infection with two *O*. *tsutsugamushi* genotypes, whereas the rest of the mites (two *Leptotrombidium* spp. and one *Blankaartia* spp.) showed mixed infection with three genotypes. The genotype composition and the relative abundance of each of the genotypes were determined by the number of NGS reads, or by the number of clones for those sequences amplified using the cloning technique (Table 4).

### *O*. *tsutsugamushi* genotypes in co-feeding mites

Of the 10 small mammal and rodent hosts infested with *Orientia*-infected mites, six were found to have 2–10 infected mites feeding on them at the time of collection (Table 3). Three of these animals had two *Orientia*-infected mites, while the remaining three hosts had 3, 5, and 10 *Orientia*-infected mites, respectively. The relationship between the *O*. *tsutsugamushi* genotypes and the mite genera collected from each host was then examined. The majority of infected mites were *Leptotrombidium* spp., and were collected from hosts where this genus was predominant (86.2–100%). However, when a host was infested with several mite genera, the number of infected mites proportionately reflected the relative abundance of each of the mite genera. For example, 90 mites were collected from *R*. *rattus* complex individual MS0651, and belonged to three different genera: 71 *Ascoschoengastia* spp., 16 *Lorillatum* spp., and 3 *Gahrliepia* spp. Of these 90 mites, only three were infected with *O*. *tsutsugamushi*, two of which were *Ascoschoengastia* spp. and one was *Lorillatum* spp. No infection was detected in *Gahrliepia* spp. mites, which were present on the host in the lowest numbers. The same situation occurred with mites collected from *R*. *rattus* complex individual DS024 (n = 19), which showed an almost equal prevalence of *Leptotrombidium* spp. (n = 10) and *Gahrliepia* spp. (n = 9). In this case, one infected mite was identified from each genus. Also, among mites collected from *B*. *indica* individual DS123 (n = 36), two of the infected mites were *Leptotrombidium* spp. (n = 26), while the remaining infected mite was *Blankaartia* spp. (n = 10).

**Fig 2:**
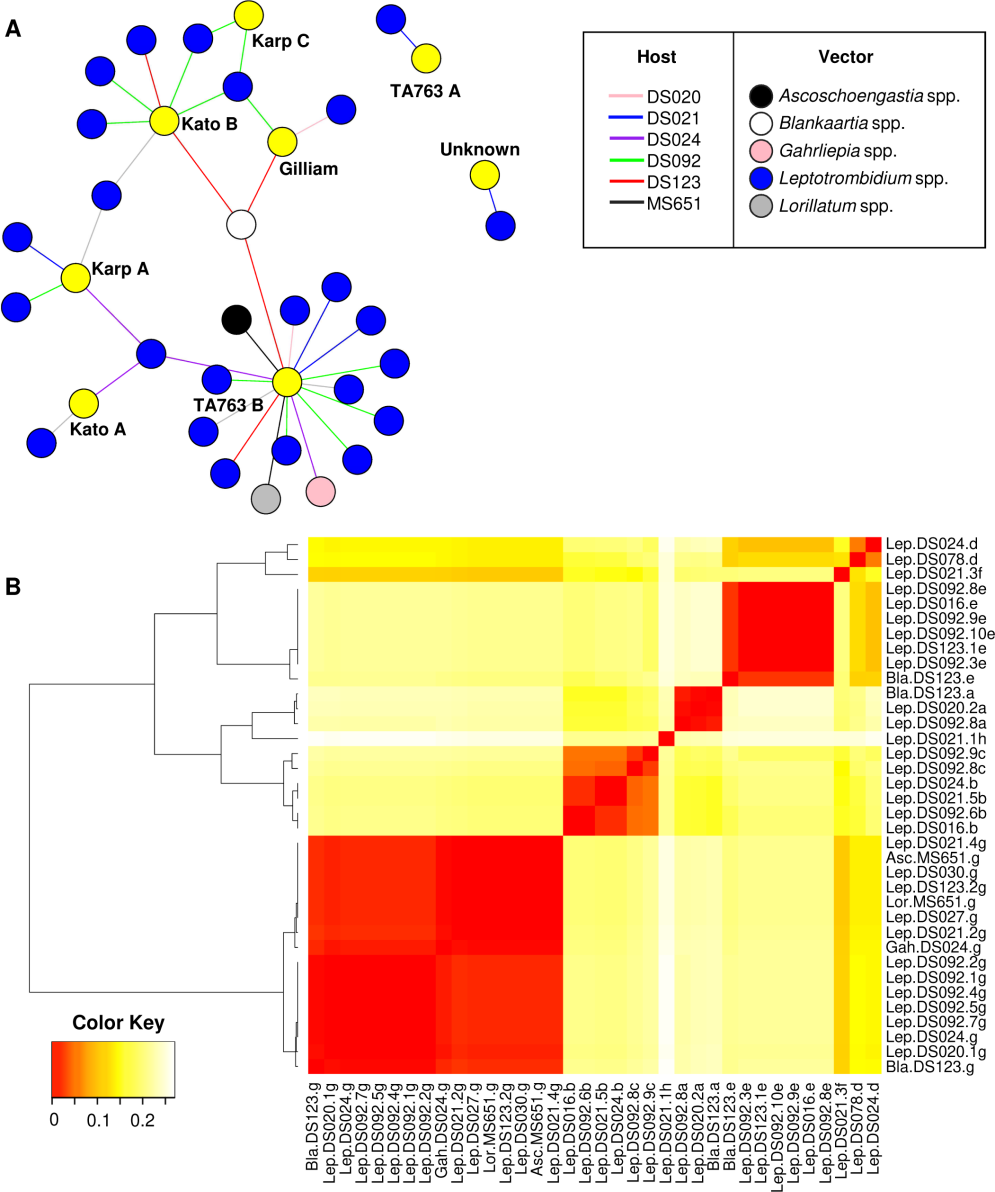
Relationship between co-feeding mites and diversity of Orientia tsutsugamushi 56-kDa TSA gene sequence-based genotypes detected in mite populations. (A) Network graph demonstrates the potential co-feeding transmission of *O*. *tsutsugamushi* genotypes (yellow circle) among infected mites on the same host (indicated by colored lines). Only hosts with multiple infected mites were included in the analysis. Hosts infested with single *O*. *tsutsugamushi*-infected mite were indicated with grey line. The graph was created in the igraph R package. (B) Heatmap demonstrating a pair-wise distance matrix of the 56-kDa TSA gene sequences among 36 *O. tsutsugamushi* genotypes identified in 28 infected mites. The heatmap was generated using the Heatmap.2 function of the gplots R package.

A network graph was generated to determine the relationships between *O*. *tsutsugamushi* genotypes detected in mites collected from the same animal host (Fig 2A). From hosts infested with 2–10 infected mites, identical *O*. *tsutsugamushi* genotypes were detected among infected mites collected from the same host (Table 3). For example, two *R*. *rattus* complex individuals (MS0651, black line; DS024, purple line in Fig 2A) each had two infected mites (MS0651: *Ascoschoengastia* and *Lorillatum* mites; DS024: *Gahrliepia* and *Leptotrombidium* mites), each of which carried the TA763 B genotype. An interesting case was observed in a *B*. *indica* rat (DS123, red line in Fig 2A) infested with three infected mites. Two of the mites (*Leptotrombidium* spp.) had the Kato B and TA763 B genotypes, respectively, while the third infected mite (*Blankaartia* spp., white circle) contained both genotypes. A similar situation was observed in another *B*. *indica* rat (DS092, green line) infested with 10 infected *Leptotrombidium* mites. Eight mites had a single infection, while two had mixed infection. Among the mites with a single infection, five shared the same TA763 B genotype and two had the Kato B genotype. The latter genotype was also detected in two infected mites with mixed infection, and both of these mites also shared the Karp C genotype. In contrast, in a common tree shrew (DS021, *T*. *glis*, blue line in Fig 2A), only two out of five infected mites (*Leptotrombidium* spp.) shared the same genotype (TA763 B). Likewise, two infected *Leptotrombidum* spp. infested on another common tree shrew (DS020, *T*. *glis*, pink line in Fig 2A) carried different *O*. *tsutsugamushi* genotypes (TA763 B and Gilliam). The heatmap shown in Fig 2B outlines the nucleotide sequence distance matrix of the 36 *O*. *tsutsugamushi* 56-kDa TSA gene genotypes detected in 28 infected mites. Alignment and identity analysis of the 56-kDa TSA gene sequences belonging to the same genotypes revealed a high level of similarity for each of the genotypes, with percent identities ranging from 98–100%, except for the Kato A genotype, where only 89% identity was observed.

## Discussion

This study is the first to provide evidence of the co-existence of multiple *O*. *tsutsugamushi* genotypes in field-collected trombiculid mites using NGS based on the 56-kDa TSA gene (variable domains I–III). These findings build on our previous research showing that different *O*. *tsutsugamushi* strains can co-exist in laboratory-reared *Leptotrombidium* mites maintained in an ABSL-3 facility [35, 36]. Co-existing genotypes, representative of mixed infections, occurred in five out of 28 infected mites (17.9%) examined in the current study. In addition, identical *O*. *tsutsugamushi* genotypes were detected among co-feeding trombiculid mites, which may indicate co-feeding transmission. Co-feeding transmission of *O*. *tsutsugamushi* occurs among laboratory-reared infected and uninfected mites (*Leptotrombidium* spp. and *Blankaartia* spp.) that had co-fed on ICR mice [40]. The current study suggests that co-feeding transmission may also occur naturally among field-collected trombiculid mites feeding on the same animal host, leading to mixed infections or multiple infected mites carrying identical *O*. *tsutsugamushi* genotypes. This conclusion is supported by two main observations. First, a single *O*. *tsutsugamushi* genotype was detected in multiple mites belonging to different genera. Second, one *O*. *tsutsugamushi* genotype was detected in a mite with mixed infection, with the same genotype also found in a co-feeding mite with single infection (Table 4; Fig 2A). For example, *B*. *indica* hosts DS092 and DS123 were infested with 10 and 3 infected mites, respectively, all of which shared similar/identical *O*. *tsutsugamushi* genotypes in both single and mixed infections. In addition, the majority of mites with mixed infection (three *Leptotrombidium* spp., one *Blankaartia* spp.) were found on hosts infested with more than two infected mites. Thus, a higher number of infected mites per host increases the likelihood of an uninfected mite acquiring *O*. *tsutsugamushi* during co-feeding [40].

Among the rodents collected in the current study, mites were mostly collected from the inner earlobe, and some hosts were heavily infested (as many as 181 mites/host). Only a small number of mites were found on the soft tissues of the ventral and genital areas. From previous field experience, clusters of mites are rarely found on the ventral surfaces of rodents, possibly because rodents are able to groom these areas; whereas, they cannot groom inside their ears and clusters of mites are able to form. In contrast, mites on the common tree shrew (*T*. *glis*) were predominantly found on the ventral and genital areas, with few mites in the inner earlobe. This is probably because the skin in the inner ear of shrews is very thick compared to rodents, and is not conducive to chigger feeing. The observation that mites were more widely dispersed on shrews and did not feed in tight clusters within the inner earlobe could be one possible hypothesis to explain why infected mites on shrews did not have identical *O*. *tsutsugamushi* genotypes, and showed no evidence of co-feeding transmission.

Larval mites usually feed once on a rodent host for a period of 2–5 days [40, 69]. Mites on rodents were most often observed feeding in tight clusters in the inner earlobe, supporting the likelihood that if there was an infected mite, the close proximity and duration of feeding would provide the opportunity for co-feeding transmission of *O*. *tsutsugamushi* from infected to naïve mites. Similar observations have been made for *Rhipicephalus sanguines* ticks, where co-feeding transmission efficiency of *R*. *conorii* is greatly increased by close proximity of infected and naïve ticks [47]. Therefore, we speculate that co-feeding transmission could be another mode of *O*. *tsutsugamushi* transmission, in addition to transstadial and transovarial transmission processes, and play a significant role in maintaining *O*. *tsutsugamushi* in mite populations in nature.

In the high-prevalence area (Pang Nga Province), *Leptotrombidium* species were the most abundant mites, and were mainly associated with *R*. *rattus* complex and *B*. *indica* rats. Moreover, *Leptotrombidium* was the most commonly infected mite genus in this area, which is consistent with previous studies showing that *Leptotrombidium* spp. are still the main vectors for scrub typhus transmission [23, 70–73]. Interestingly, our findings confirm a previous study that used direct immunofluorescence assays (DFA) to show that *O*. *tsutsugamushi* was present in *Blankaartia acuscutellaris* mites collected from rodents in central Thailand [27]. However, further investigation by the same group failed to isolate *O*. *tsutsugamushi* in laboratory mice from field-collected *B*. *acuscutellaris* [26]. The authors suggested that the detection of *O*. *tsutsugamushi* by DFA in *B*. *acuscutellaris*, which has been reported previously by Tanskul et al. [27], might be a result of co-feeding transmission or acquisition of the bacterium from a systemically infected host, as these modes of transmission have been shown to occur in an experimental mice and laboratory-reared mites [40]. However, our current study used molecular detection methods to identify *O*. *tsutsugamushi* infection not only in *Bankaartia* spp., but also in *Ascoschoengastia* spp., *Gahrliepia* spp., and *Lorillatum* spp. Additionally, some of these genera were found feeding in the earlobe in close proximity to *Leptotrombidium* spp. Acquisition from a systemically infected host could be excluded, as all hosts infested with *O*. *tsutsugamushi-*positive mites were negative for *O*. *tsutsugamushi* infection by molecular assay. Therefore, the presence of *O*. *tsutsugamushi* in these mites could be a result of co-feeding transmission from infected *Leptotrombidium* mite(s) feeding in the close proximity on the same host. This could explain the finding of an identical *O*. *tsutsugamushi* genotype in *Gahrliepia* spp. and *Leptotrombidium* spp. mites on *R*. *rattus* complex individual DS024, and in a *Blankaartia* spp. mite on *B*. *indica* individual DS123. However, the *Blankaartia* mite collected from DS123 also carried the Gilliam genotype, which was not detected in its co-feeding *Leptotrombidium* mites. So, the question remains of how the *Blankaartia* mite acquired the Gilliam genotype. A similar observation was made among *Ascoschoengastia* and *Lorillatum* mites co-feeding on *R*. *rattus* complex MS651, where both mites carried TA763 B genotype bacteria. However, because all of these conclusions are based solely on data from field-collected mites, where transmission could not be monitored, other unknown factors could have influenced the results. For example, Gilliam genotype detected in *Blankaartia* mite infested on host DS123 may have been present in the mite prior to attachment to this host (i.e. it has been maintained in the mite via transovarial transmission). Further study is therefore needed to investigate whether other genera are capable of transmitting *O*. *tsutsugamushi* in nature, and to confirm whether co-feeding transmission results in mixed infection of *O*. *tsutsugamushi* genotypes. Such co-feeding transmission studies can be conducted using laboratory-reared infected mites and animal models, allowing for more controlled experimentation.

It is worth noting that an abundance of *Leptotrombidium* mites or a higher chigger index does not necessarily directly correspond to an increased prevalence of *O*. *tsutsugamushi* in mite populations. This was the case in Chumphon Province, which had the second highest abundance of mites, and where the majority of mites were *Leptotrombidium* spp. However, no infected mites were detected in this area. In addition, two infected mites (*Ascoschoengastia* spp. and *Lorillatum* spp.) were collected from a *R*. *rattus* complex in Sisaket Province; however, no *Leptotrombidium* spp. mites were collected from animals captured in this area. Based on our study data, Pang Nga Province could be considered a hotspot for scrub typhus transmission within mite populations, as multiple infected mites were usually found on each animal host. Interestingly, during the study period, 211 human cases of scrub typhus were reported in Pang Nga Province by the Thai Ministry of Public Health. The current study also revealed highly diverse within-host *O*. *tsutsugamushi* genotypes, with as many as three different *O*. *tsutsugamushi* genotypes found in individual mites. The ecological dynamics of multiple *O*. *tsutsugamushi* genotypes in one host could drive pathogen evolution/diversification in the vector. This may be consistent with the hypothesis that genetic recombination among different *O*. *tsutsugamushi* genotypes can occur in the mite vector [74].

## Supporting information

S1 Fig Phylogenetic analysis of *Orientia tsutsugamushi* 56-kDa type-specific antigen gene sequences for each of the genotypes (consensus sequences from next-generation sequencing reads or cloning) detected in field-collected trombiculid mites and reference sequences retrieved from the GenBank database. A maximum likelihood tree was constructed using the GTR+G model of nucleotide substitution in the MEGA 6 program with bootstrapping (1000 replicates). Percent abundance of each genotype detected in individual mites is indicated in parenthesis after each sequence.
(TIF)

S2 Fig *Orientia tsutsugamushi* prevalence in small mammals and trombiculid mites collected from several provinces in Thailand (2012–2016). (PDF)

S1 Table List of *Orientia tsutsugamushi* 56-kDa type-specific antigen gene reference sequences used as local references in Map Reads to Reference analysis. Analysis was performed using CLC Genomics Workbench.
(PDF)

S2 Table Characterization of *O*. *tsutsugamushi* genotypes (based on 56-kDa TSA gene, variable domains I-III) from three rodents collected from Phang Nga province by cloning. A nucleotide distance matrix was generated using “DNADist DNA Distance Matrix” in BioEdit.
(PDF)
