# Supplementary figures and images for: Heterogeneity of Orientia tsutsugamushi genotypes in field-collected trombiculid mites from wild-caught small mammals in Thailand
Source: PLoS Negl Trop Dis. 2018 Jul 16;12(7):e0006632. doi: 10.1371/journal.pntd.0006632 (PMC6062101; doi:10.1371/journal.pntd.0006632)

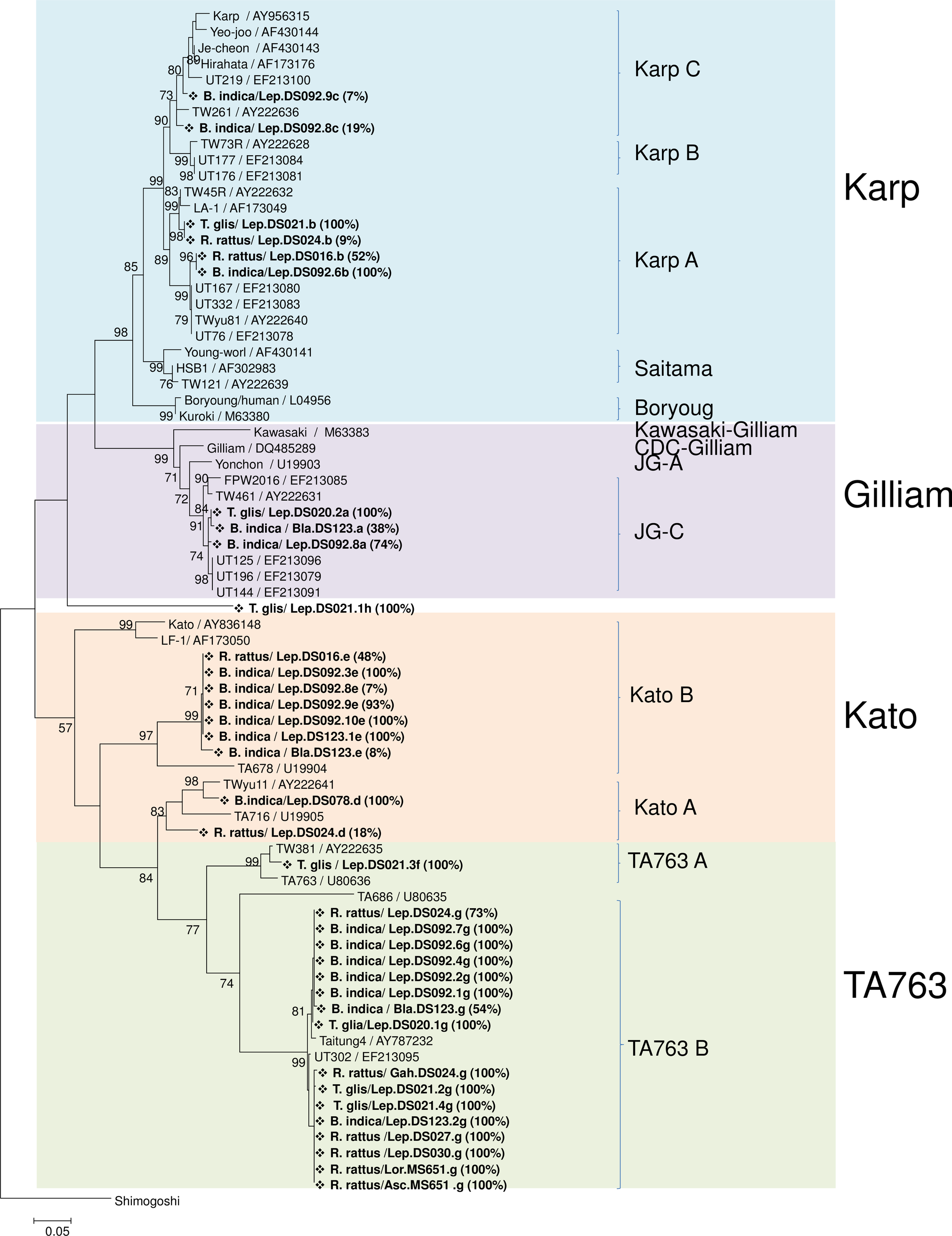

Supplement: S1 Fig — A maximum likelihood tree was constructed using the GTR+G model of nucleotide substitution in the MEGA 6 program with bootstrapping (1000 replicates). Percent abundance of each genotype detected in individual mites is indicated in parenthesis after each sequence. (TIF) [file pntd.0006632.s001.tif]

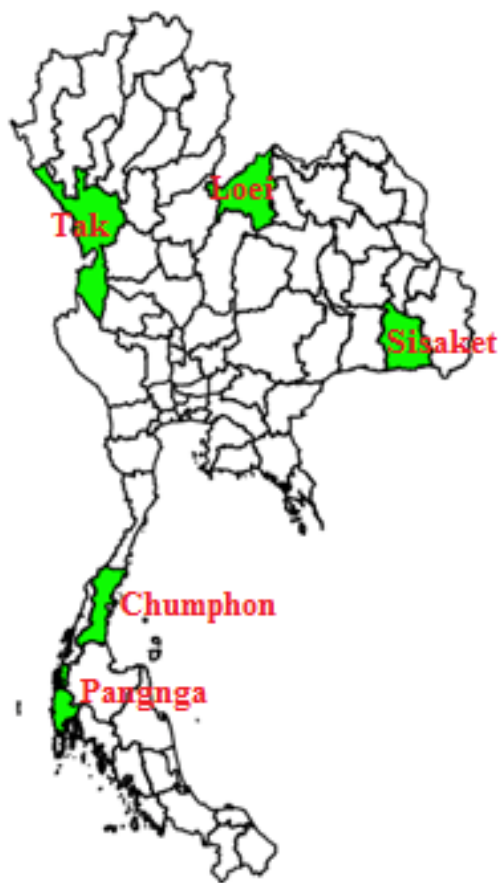

Collection sites

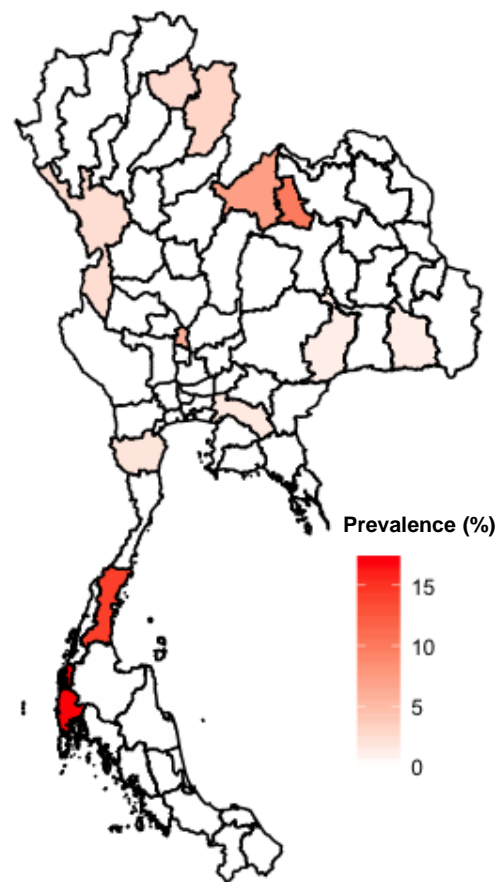

*O. tsutsugamushi* prevalence rate (%) in small mammals and chiggers

Supplement: S2 Fig — (PDF) [file pntd.0006632.s002.pdf]
