# Supplementary material for: Heterogeneity of Orientia tsutsugamushi genotypes in field-collected trombiculid mites from wild-caught small mammals in Thailand
Source: PLoS Negl Trop Dis. 2018 Jul 16;12(7):e0006632. doi: 10.1371/journal.pntd.0006632 (PMC6062101; doi:10.1371/journal.pntd.0006632)
Supplement: S1 Table — Analysis was performed using CLC Genomics Workbench. (PDF) [file pntd.0006632.s003.pdf]

**S1 Table. List of *Orientia tsutsugamushi* 56-kDa type-specific antigen gene reference sequences used as local references in Map Reads to Reference analysis.** Analysis was performed using CLC Genomics Workbench.

| Isolate name | Isolate host | Location | Year of isolation | Accession no. | Group classified/genotype |
|---|---|---|---|---|---|
| Gilliam | Human | Myanmar | 2002 | DQ485289 | Gilliam |
| Karp | Human | New Guinea | 1943 | AY956315 | Karp/Karp C |
| Kato | Human | Japan | 1955 | AY836148 | Kato/Kato B |
| Kawasaki | Human | Japan | 1981 | M63383 | Gilliam/Kawasaki |
| Kuroki | Human | Japan | 1981 | M63380 | Karp/Boryong |
| LA-1 | *Leptotrombidium arenicola* | Malaysia | 1993 | AF173049 | Karp/Karp A |
| TA678 | *Rattus rattus* | Thailand | 1963 | U19904 | Kato/Kato B |
| TA686 | *Tupaia glis* | Thailand | 1963 | U80635 | Shimokoshi |
| TA716 | *Menetes berdmorei* | Thailand | 1963 | U19905 | Kato/Kato A |
| TA763 | *Rattus rajah* | Thailand | 1963 | U80636 | TA763/TA763 A |
| Taitung-4 | Human | Taiwan | 2004 | AY787232 | TA763/TA763 B |
| TW45R | *Rattus losea* | Taiwan | 1999 | AY222632 | Karp/Karp A |
| UT76 | Human | Thailand | 2003 | EF213078 | Karp/Karp A |
| UT177 | Human | Thailand | 2004 | EF213084 | Karp/Karp B |
| UT302 | Human | Thailand | 2004 | EF213095 | TA763/TA763 B |
| UT332 | Human | Thailand | 2005 | EF213083 | Karp/Karp A |
| Young-worl | Human | South Korea | N/A | AF430141 | Karp/Saitama |
