# Supplementary material for: Heterogeneity of Orientia tsutsugamushi genotypes in field-collected trombiculid mites from wild-caught small mammals in Thailand
Source: PLoS Negl Trop Dis. 2018 Jul 16;12(7):e0006632. doi: 10.1371/journal.pntd.0006632 (PMC6062101; doi:10.1371/journal.pntd.0006632)
Supplement: S2 Table — A nucleotide distance matrix was generated using “DNADist DNA Distance Matrix” in BioEdit. (PDF) [file pntd.0006632.s004.pdf]

S2 Table. Characterization of *O. tsutsugamushi* genotypes (based on 56-kDa TSA gene, variable domains I-III) from three rodents collected from Phang Nga province by cloning. A nucleotide distance matrix was generated using "DNADist DNA Distance Matrix" in BioEdit.

			References (% Identity)											
			**Karp A**	**Karp A**	**Karp B**	**Karp C**	**Saitama**	**Boryong**	**Kawasaki-Gilliam**	**JG-C**	**Kato A**	**Kato B**	**TA763A**	**TA763B**
**Rodent ID**	**Host species**	**Numbers of clone**	**UT332**	**TW45R**	**UT177**	**Karp**	**Youngworld**	**Kuroki**	**Kawasaki**	**Gilliam**	**TA716**	**Kato**	**TA763**	**UT302**
**DS0083**	**R. rattus complex**	**26**	99.70	87.30	84.60	85.50	86.80	80.50	65.60	71.30	62.00	62.10	69.10	59.50
**DS0094**	***R. rattus*** **complex**	**30**	99.70	87.30	84.60	85.50	86.80	80.50	65.60	71.30	62.00	61.70	69.10	59.50
**DS0114**	***B. indica***	**28**	87.10	99.70	93.00	93.20	88.40	89.50	69.20	75.30	64.80	68.60	68.00	62.80
